# A BCI-Based Study on the Relationship Between the SSVEP and Retinal Eccentricity in Overt and Covert Attention

**DOI:** 10.3389/fnins.2021.746146

**Published:** 2021-12-14

**Authors:** Yajun Zhou, Li Hu, Tianyou Yu, Yuanqing Li

**Affiliations:** ^1^Center for Brain Computer Interfaces and Brain Information Processing, South China University of Technology, Guangzhou, China; ^2^Guangzhou Key Laboratory of Brain Computer Interaction and Application, Guangzhou, China

**Keywords:** covert attention, overt attention, electroencephalography, steady-state visual evoked potential, brain-computer interface

## Abstract

Covert attention aids us in monitoring the environment and optimizing performance in visual tasks. Past behavioral studies have shown that covert attention can enhance spatial resolution. However, electroencephalography (EEG) activity related to neural processing between central and peripheral vision has not been systematically investigated. Here, we conducted an EEG study with 25 subjects who performed covert attentional tasks at different retinal eccentricities ranging from 0.75° to 13.90°, as well as tasks involving overt attention and no attention. EEG signals were recorded with a single stimulus frequency to evoke steady-state visual evoked potentials (SSVEPs) for attention evaluation. We found that the SSVEP response in fixating at the attended location was generally negatively correlated with stimulus eccentricity as characterized by Euclidean distance or horizontal and vertical distance. Moreover, more pronounced characteristics of SSVEP analysis were also acquired in overt attention than in covert attention. Furthermore, offline classification of overt attention, covert attention, and no attention yielded an average accuracy of 91.42%. This work contributes to our understanding of the SSVEP representation of attention in humans and may also lead to brain-computer interfaces (BCIs) that allow people to communicate with choices simply by shifting their attention to them.

## 1. Introduction

Our eyes are constantly subjected to a complete image of the visual world that contains large amounts of information than we can process at once. One mechanism that limits these processing resources to the most relevant aspects of the environment is selective attention, which optimizes our visual processing through trade-offs in which the representations of attended locations or features of our environment are enhanced, while those of unattended locations or features are diminished. This attention can be further categorized as overt when we move our eyes to a relevant location and the focus of attention coincides with the movement of the eyes or as covert when attention is deployed to a relevant location without accompanying eye movements (Carrasco and Yeshurun, 2009).

Many studies have behaviorally examined the influence of eccentricity (or retinal eccentricity, refers to the angular distance when the light of the object enters the eye and approaches peripheral vision, Carrasco et al., 1995) on the visual response during covert attention. Posner (1980) reported that the behavioral response was slower and less accurate when the cue targets appeared at an unattended location rather than at the attended location. Later, Trachel et al. (2015) demonstrated that subjects can perform voluntary covert attention shifts with more ambiguous directional cues. Moreover, spatial cue paradigms can be improved when multiple targets with different eccentricities are introduced during covert attention (Freeman and Simoncelli, 2011; Harvey and Dumoulin, 2011; Staugaard et al., 2016). It has also been shown that information about visual targets accrues faster at attended than at unattended locations (Carrasco and McElree, 2001; Carrasco et al., 2006).

Other studies focused on covert attention based on electrophysiological tools such as electroencephalography (EEG), magnetoencephalography (MEG) (Van Gerven et al., 2009), and functional magnetic resonance imaging (fMRI) (Perry and Zeki, 2000; Harvey and Dumoulin, 2011), which can provide more detailed information about ongoing brain activities. For example, Rihs et al. (2007) showed a strong correlation between alpha power and covert attention to stimuli in eight different orientations but located with the same eccentricity. Later, Bahramisharif et al. (2011) explored the lateralization pattern under six different eccentricity conditions, while Roijendijk et al. (2013) demonstrated that lateralization depends on the difficulty of the covert attention task. Recently, a study found that attention speeds visual processing by discovering that the latency of the evoked N2pc component is reduced for visual targets that appear at attended (overt) locations (Foster et al., 2020). However, there has been insufficient analysis of covert attention, in which targets are placed at a fixed arbitrary eccentricity. In addition, the pattern of EEG activity modulation as a function of the eccentricity of the targets to which one should attend remains unknown.

Covert decoding results can also be used in some brain-computer interface (BCI) systems, and the N2pc component can also be evoked in a covert shift attention task to decode one of four targets presented in the left and right visual fields (Freeman and Simoncelli, 2011). Studies have also shown that alpha modulation patterns can be classified while shifting to spatial directions (Van Gerven and Jensen, 2009; Van Gerven et al., 2009). Tonin et al. (2012) demonstrated that alpha subbands generated during covert attention can be exploited to enhance BCI classification accuracy. Moreover, Gerven and Jensen [19] also showed the possibility of detecting four directions at 90° angles with up to 70% accuracy using the covert attention paradigm and MEG recording. Finally, two pieces of evidence suggest that steady-state visual evoked potentials (SSVEPs), another important paradigm in a BCI system, can be applied to decode targets with classification accuracies of up to 79% (Kelly et al., 2005) and 72.6% (Zhang et al., 2010) in a covert attention experiment involving the application of two flicker stimuli (i.e., eccentricity).

This study aims to examine the relationship between EEG modulation and covert attention with different retinal eccentricities, compare the EEG characteristics between covert and overt attention and investigate the decoding performance of overt and different covert attention tasks, as well as tasks without attention. Specifically, we recorded SSVEPs with a single stimulus frequency (i.e., 7.5 Hz) to examine the resonance phenomenon of subjects' brain activities during covert and overt attention tasks. A feature extraction method based on filter bank canonical correlation analysis (FBCCA) for SSVEP analysis revealed a significant negative relationship between the synthetic correlation parameter during covert attention and the eccentricity. We then calculated the power spectral density (PSD) and intertrial phase coherence (ITPC) to compare the EEG recordings between overt attention and different covert attention. Furthermore, the results showed a satisfactory decoding performance for three classes of attention (overt, covert, and none), achieving an average accuracy of 91.42%.

## 2. Materials and Methods

### 2.1. Subjects

Twenty-seven healthy subjects (19 males and 6 females, two subjects were excluded because of poor performance of fixing their eyes or head movements during the attention period; ages range: 21–38 years old) were recruited to participate in the experiment. All of them provided informed consent for their data to be published. This study was approved by the Ethics Committee of Sichuan Provincial Rehabilitation Hospital (approval number: CKLL-2018008), which is our cooperating institution. All participants had normal or corrected-to-normal vision.

### 2.2. Stimuli and Procedure

EEG data were recorded from 64 channels using electrodes placed according to the standard 10-20 electrode system using a Synamps2 system (Neuroscan, Inc.) with a sampling rate of 250 Hz. The electrodes placed on the forehead (GND) and right mastoid (A2) were used as the ground and reference electrodes, respectively. The 24 channels in the occipital region, including CP1, CPz, CP2, P7, P5, P3, P1, Pz, P2, P4, P6, P8, PO7, PO5, PO3, POz, PO4, PO6, PO8, CB1, O1, Oz, O2, and CB2 based on the standard positions in the 10-20 system, were chosen for further analysis since we were only concerned with vision-related activity (Chen et al., 2015b). The impedances of the electrodes were maintained below 10 kΩ. Eye movements were controlled for with two additional channels that recorded the horizontal electrooculogram. The experiments were presented on an LCD monitor at 60 Hz and a screen resolution of 1920 × 1080. Participants were seated in a dimly lit room and kept their eyes approximately 60 cm from the screen.

**Figure 1:**
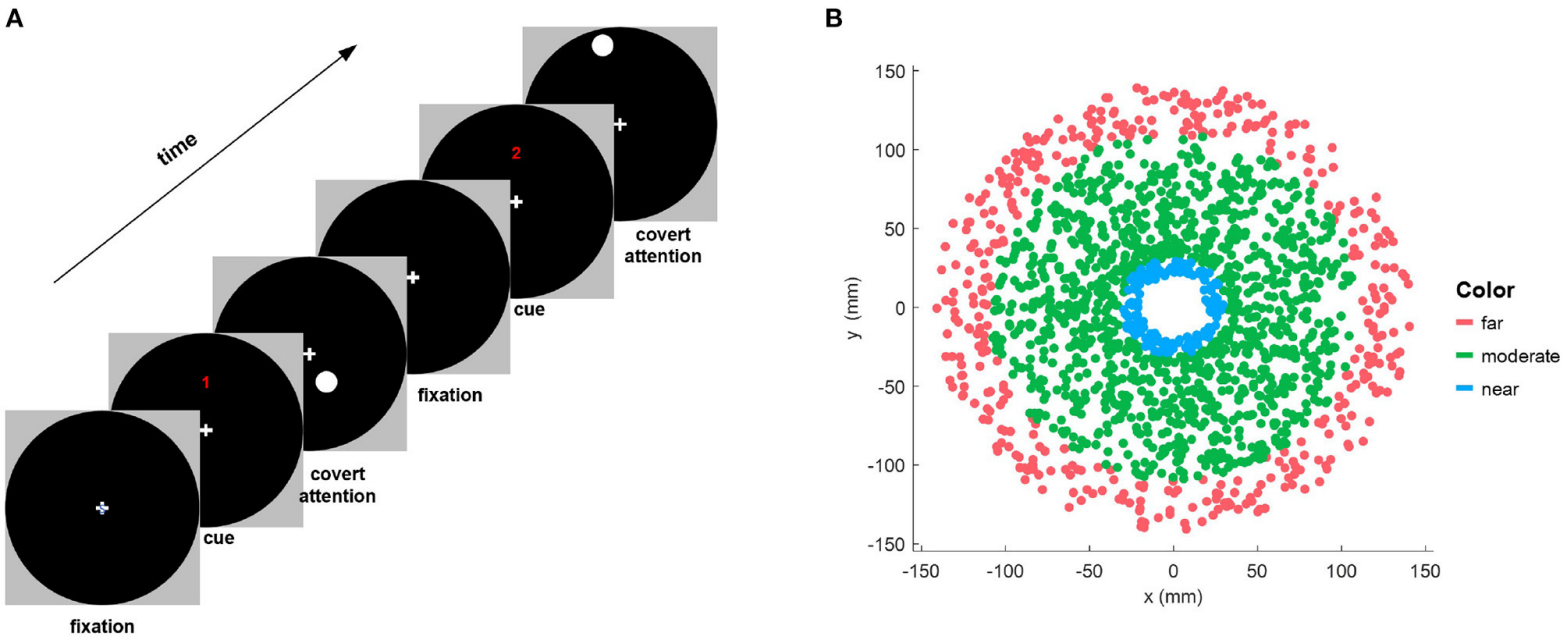
**(A)** Illustration of the experimental paradigm for one trial of the covert attention task. Each run of trials consisted of a symbolic cue telling the subjects to fixate on a central cross on the screen, followed by a visual stimulation period in which the subjects covertly shifted their attention to a flickering circle displayed at different positions. **(B)** Distribution of the locations of stimulus targets that are shown on the black circular background of the left column for all subjects. The red, green, and blue dots indicate targets of different types of eccentricity (far, moderate, and near, respectively).

First, 70 trials were presented during the covert attention phase. As depicted in Figure 1A, at the beginning of each trial, the participants were instructed to fixate on a cross at the center of the screen. Then, a numerical cue appeared for 2 s on the top of the cross, indicating the upcoming 8-s period of covert attention, in which the participants had to shift their attention to a stimulus target that flickered at a frequency of 7.5 Hz while strictly maintaining focus on the center cross. It should be noted that familiar with the instruction before the experiments especially the covert attention experiment was necessary for all participants. In our approach, in order to maintain the focusing state and during stimulation, 2 s of preparation was also provided for participants to move their gaze to the central cross on the screen before the onset of peripheral stimulation on each trial. Besides, the severe eye movements were detected and the corresponding trials were rejected by manual proofreading of the recorded EOG signals. Moreover, we also monitored the head movement in the thole procedure of each trial simultaneously. The participants need to provide feedback of their concentrate performance when the experiments were completed. The shape of the stimulus target was a circle of size 0.75° (i.e., a radius of 7.81 mm) whose position was randomly distributed in a circular area of size 13.90° (i.e., a radius of 148 mm).

All subjects were also required to participate in a controlling experiment (i.e., overt attention) involving 20 trials for each subject. The only difference with the covert attention experiment trials was that the fixation cross was removed; instead, each participant was asked to fix his or her attention on the flickering stimulus circle during each overt attention trial, in which the duration of stimulus presentation was kept the same (i.e., 8 s). Subsequently, an additional 20 trials without attentional requirements were collected for each participant for the subsequent classification analysis. Specifically, people need to remain idle state (i.e., they were allowed to freely blink as usual, and perform some basis physiological activities but avoid vigorous body movements) during the experiments of no attention.

A 2-s interval was presented between continuous trials for both the covert and overt attention experiments, during which no flickering circles were displayed on the screen. The onset of each trial was cued by a transitory text prompt. During the attention period of each trial, the subjects were instructed to refrain from blinking as much as possible. To keep the subjects engaged, they were required to count every second until the end of each stimulus period.

To assess the SSVEP response in the covert attention task for different degrees of spatial distance, we divided the eccentricities into three levels as follows. Eccentricities not greater than 3° (i.e., Euclidean distance ≤ 31.45*mm* in this study) was defined as “near,” while eccentricities not less than 10° (i.e., Euclidean distance ≥105.8*mm* in this study) was defined as “moderate,” and the remaining eccentricities were defined as “far” (Figure 1B).

### 2.3. EEG Acquisition and Preprocessing

Raw EEG signals were acquired from the 64 channels using electrodes placed according to the standard 10-20 electrode system using a SynAmpsRT amplifier (Neuroscan Compumedics, Singen, Germany) at a sampling frequency of 250 Hz. The EEG signals were bandpass filtered between 4 and 25 Hz using a 4*th* − *order* Butterworth filter prior to further analysis. Trials containing signal artifacts corresponding to behaviors such as jaw clenching, muscle movement, and electrode disconnections were removed by visual inspection. Next, independent component analysis (ICA) was performed to remove blinks and eye movements using the FieldTrip toolbox (Version 20201021, http://www.fieldtriptoolbox.org) with default parameters. Components with upper 70% confidence labeled as eye artifacts were then removed. All analyses were performed in Matlab.

### 2.4. Feature Extraction

This study employed the extended canonical correlation analysis (CCA)-based method, using the multiple trials of overt attention as individual calibration signals to extract the metrical features for the test signals during covert attention (Nakanishi et al., 2014, 2015; Chen et al., 2015b). Here, with ***Y*** representing the sinusoidal template data (Chen et al., 2015b), the individual calibration signals for the *n*th subband component were denoted by a three-way tensor $\hat{\chi }\in {\mathbb{R}}^{N_{n}\times N_{c}\times N_{s}}$, and the test signals of each trial during covert attention were denoted by a four-way tensor $X\in {\mathbb{R}}^{N_{t}\times N_{n}\times N_{c}\times N_{s}}$. Note that *N*_*t*_ indicates the number of stimulation trials during the covert attention experiment, *N*_*n*_ indicates the number of subband components, *N*_*c*_ is the number of channels and *N*_*s*_ is the number of sampling points in each trial. Filter bank analysis was applied to decompose the SSVEPs into subband components so that the harmonic components could be extracted more efficiently (Chen et al., 2015a). In practice, Butterworth infinite impulse response (IIR) bandpass filters that shared the same upper-bound frequency (30 Hz) but had different lower-bound frequencies (i.e., for the *n*th subband component ***X***_*n*_, the lower-bound frequency is 4+(*n*−1)×6 Hz) were used to extract the harmonic components (i.e., *N*_*n*_ = 3 in this study) from the test EEG data ***X***. Next, the following weight vectors were used as spatial filters to enhance the signal-to-noise ratio (SNR) of the SSVEPs: (i) ***U***_***X***_*n*__ and ***V***_***X***_ were calculated using the *n*th subband component of the test EEG data epoch ***X***_*n*_ and the sine-cosine template data epoch ***Y***. (ii) $U_{X_{n}{\hat{X}}_{n}}$ and $V_{X_{n}{\hat{X}}_{n}}$ were calculated using ***X***_*n*_ and the *n*th subband component of the individual calibration data, ${\hat{X}}_{n}$. (iii) $U_{{\hat{X}}_{n}Y}$ and $V_{{\hat{X}}_{n}Y}$ were calculated from ${\hat{X}}_{n,k}$ and ***Y***.

Then, a vector of correlation coefficients was obtained using these three pairs of weight vectors (Chen et al., 2015b; Nakanishi et al., 2015), as shown below:


(1) $$\begin{array}{l} r_{n}=[\begin{array}{c} r_{n}(1)\\ r_{n}(2)\\ r_{n}(3)\\ r_{n}(4) \end{array}]\\ \;=[\begin{array}{c} \rho (X_{n}^{T}U_{X_{n}Y},Y^{T}V_{X_{n}Y})\\ \rho (X_{n}^{T}U_{X_{n}{\hat{X}}_{n}},{\hat{X}}_{n}^{T}V_{X_{n}{\hat{X}}_{n}})\\ \rho (X_{n}^{T}U_{X_{n}Y},{\hat{X}}_{n}^{T}U_{X_{n}Y})\\ \rho (X_{n}^{T}U_{{\hat{X}}_{n}Y},{\hat{X}}_{n}^{T}U_{{\hat{X}}_{n}Y}) \end{array}],\; \end{array}$$

where ρ(***a***, ***b***) denotes the correlation coefficient between ***a*** and ***b***. Note that both ***Y*** and ${\hat{X}}_{n}$ are previously determined data epochs associated with the *k*th target. Thus, the feature of the *n*th subband of the test data epoch ***X***_*n*_ associated with the *k*th target was defined in terms of ***r***_*n*_:


(2) $$\begin{array}{l} \rho_{n}=\overset{4}{\underset{i=1}{\sum }}sign\left(r_{n}\left(i\right)\right)\cdot r_{n}{\left(i\right)}^{2}, \end{array}$$

where *sign*() is used to preserve the discriminating information because negative correlation coefficients might exist between the two signals being compared. Furthermore, a weighting coefficient *w*(*n*) was applied to each ρ_*n,k*_ because the SNR of the harmonic components of the SSVEP decreases as the evoked frequency increases. The weight coefficients are defined as follows:


(3) $$\begin{array}{l} w\left(n\right)=n^{-a}+b,n\in \left[\begin{array}{c} 1\;N \end{array}\right], \end{array}$$

where *a* and *b* are constants determined using a grid-search method during offline analysis (1 and 0, respectively, in this study). Finally, the synthetic correlation parameter ***ρ*** to specific eccentricity of each attentional trial associated with test data ***X***, is defined as a weighted square sum of the correlative features ***ρ***_*n*_ of all subbands (Chen et al., 2015b; Nakanishi et al., 2015) as follows:


(4) $$\begin{array}{l} \rho =\overset{N_{n}}{\underset{n=1}{\sum }}w\left(n\right)\cdot \rho_{n}^{2}. \end{array}$$

### 2.5. Topographical and Time-Frequency Analysis

The PSD of the EEG data was calculated by performing fast Fourier transform. The fundamental and two harmonic components of the stimulative frequency were selected as the frequencies of interest (i.e., 7.5 Hz, 15 Hz and 22.5 Hz) during the calculation. Moreover, the ITPC was calculated to visualize the trial-by-trial variations in the time-frequency domain (Yu et al., 2018; Gui et al., 2020). Specifically, the single-trial EEG data were transformed into the frequency domain from 4 to 25 Hz in steps of 0.5 Hz using wavelet transform with a sliding time window from 0 to 8 s in steps of 0.05 s. The coefficient of the wavelet transform was denoted as *X*_*k*_(*f*) for the *k*^*th*^ trial (*k* = 1, 2, …, *n*), and the ITPC is defined below:


(5) $$\begin{array}{l} \mathrm{ITPC}\left(f\right)=1/\text{n}{\left(\overset{\text{n}}{\underset{k=1}{\sum }}\cos\left(A_{k}\left(f\right)\right)\right)}^{2}+1/\text{n}{\left(\overset{\text{n}}{\underset{k=1}{\sum }}\sin\left(A_{k}\left(f\right)\right)\right)}^{2}, \end{array}$$

where *A*_*k*_(*f*) = ∠*X*_*k*_(*f*) is the phase information obtained from wavelet analysis.

### 2.6. Connection Pattern Analysis

In addition to the PSD, network patterns can be used for extracting important physiological information on the way different brain regions are functionally coupled (La Rocca et al., 2014). In this study, the network construction was estimated by calculating spectral coherence (COH), which is frequently used due to its practical and intuitive interpretation. Given two EEG signals from channels *i* and *j*, the spectral coherence *Coh*_*i,j*_(*f*) for a particular frequency *f* is defined as


(6) $$\begin{array}{l} \text{Co}{\text{h}}_{i,j}\left(f\right)=\frac{|P_{i,j}\left(f\right){|}^{2}}{P_{i,i}\left(f\right)\cdot P_{j,j}\left(f\right)}, \end{array}$$

where *P*_*i,j*_(*f*) denotes the cross-spectra of the signals corresponding to channels *i* and *j*, while *P*_*i,i*_(*f*) and *P*_*j,j*_(*f*) are the respective autospectra. To construct the network, we used the 62 electrodes as network nodes and applied COH to calculate the connectivity weights for all overt and covert attention trials.

### 2.7. Decoding Analysis

**Figure 2:**
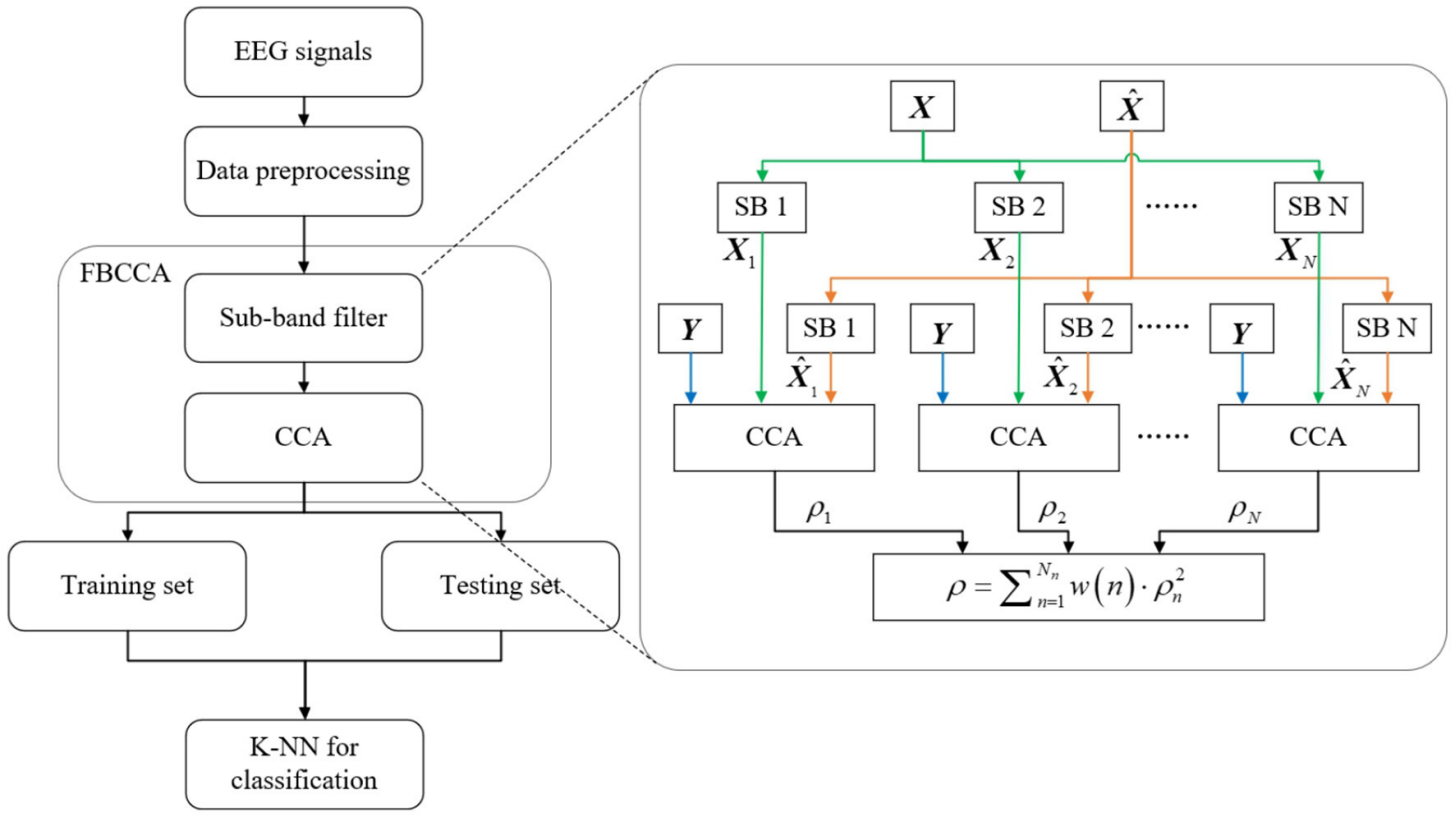
Block diagram of the classification procedure.

The overt, covert, and no attention trials were used for classification analysis separately. First, we used the above correlation values ρ calculated by FBCCA corresponding to each kind of attention trail as features for three-class classification. The k-nearest neighbors (K-NN) algorithm (Hosseini et al., 2020) was chosen as the classifier. Please refer to Figure 2 for an overview of the procedure for classification. In addition, the same classifier was used to identify which type of covert attention the subjects were attending (i.e., near, moderate, and far), with the correlation values corresponding to all three types of covert trials being selected as features. We conducted 10-fold cross-validation (i.e., 90% of the data were used as a training set and the remaining 10% of the data as a test set) to assess the classification performance for each classification and each subject. The statistical significance of the classification accuracy was evaluated through the Wilcoxon signed-rank test.

## 3. Results

### 3.1. Result III: SSVEP Response With Eccentricity

**Figure 3:**
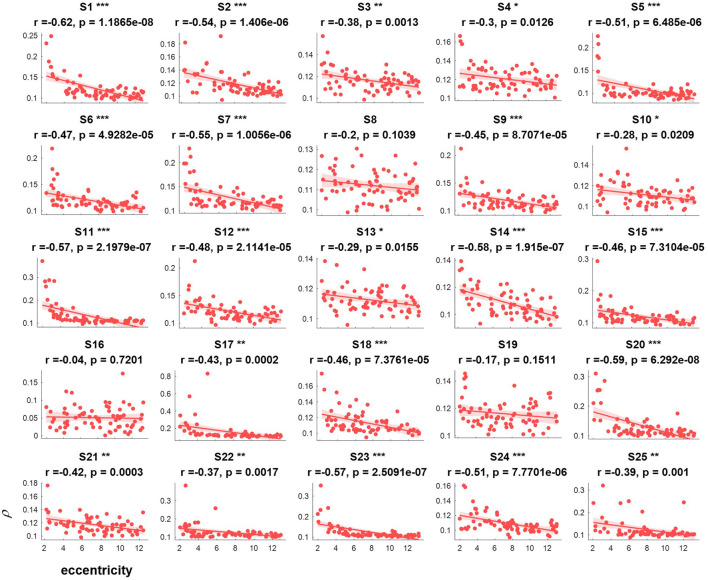
The relevance of each subject between the synthetic correlation parameter ρ in the covert attention experiment with eccentricity represented as Euclidean distance. For each specific eccentricity, the ρ was calculated using the corresponding trial with a data length of 8 s. The horizontal and vertical axes show the eccentricity and the parameter for evaluating the SSVEP response, respectively. Pearson's correlation coefficient P is noted for each plot. For each subject, the number of samples was equal to the number of covert trials. The solid lines represent the least-squares fits for the correspondingly colored data, with shading showing the 95% confidence interval (CI). *, **, and *** indicate *p* < 0.05, *p* < 0.01, and *p* < 0.001, respectively.

**Figure 4:**
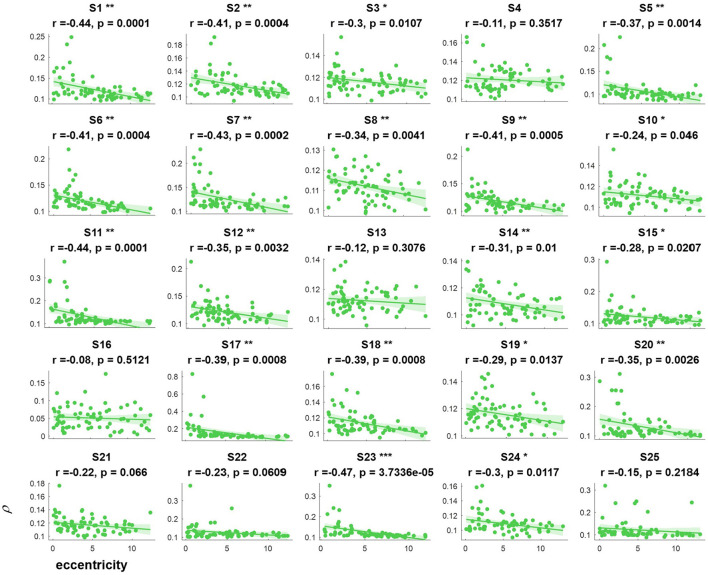
The relevance of each subject between the synthetic correlation parameter ρ in the covert attention experiment with eccentricity represented as the horizontal distance. *, **, and *** indicate *p* < 0.05, *p* < 0.01, and *p* < 0.001, respectively.

**Figure 5:**
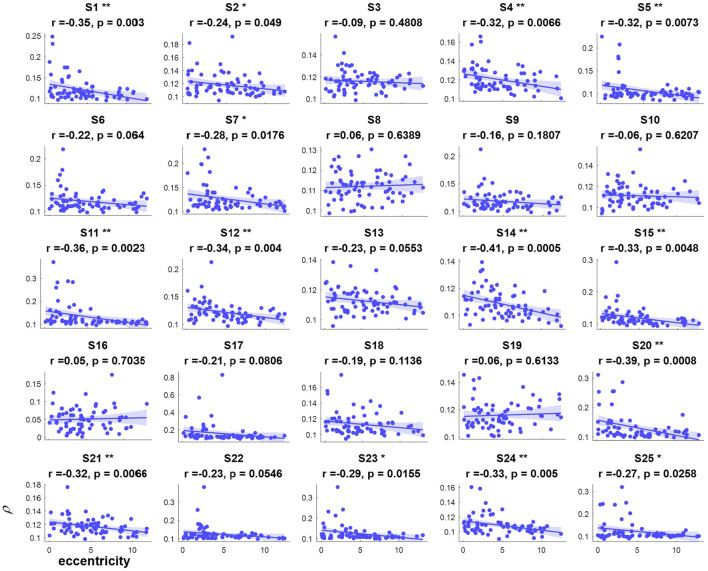
The relevance of each subject between the synthetic correlation parameter ρ in the covert attention experiment with eccentricity represented as the vertical distance. *, ** indicate *p* < 0.05, *p* < 0.01, respectively.

The correlation values and p values of the statistical tests for each subject are given in Figure 3. Furthermore, to investigate the subjects' SSVEP response with eccentricity under different visual angles, we also compared the relationship between the synthetic correlation parameter ρ and both the horizontal distance (Figure 4) and vertical distance (Figure 5). In general, the subjects generated similar characteristics of SSVEPs (i.e., across subjects, the parameter in the covert attention condition was significantly correlated with visual target eccentricity).

For the majority of subjects (except for S8, S16, and S19, Figure 3), there were negative and significant associations between the parameter in the covert attention task and the eccentricity as represented using the Euclidean distance. In addition, only six (S4, S13, S16, S21, S22, and S25, Figure 4) and eleven (S3, S6, S8, S9, S10, S13, S16, S17, S18, S19, and S12, Figure 5) of all subjects did not show significant correlations with the horizontal distance or the vertical distance were applied, respectively. Therefore, for each subject, a statistically significant negative association between the feature for covert attention and eccentricity can be found if the proper eccentricity measurement was selected (i.e., calculating the eccentricity either with the Euclidean, horizontal or vertical distance). Moreover, one-way repeated measures ANOVA revealed a significant trend of group effect [*F*_(2,72)_ = 15.4, *p* < 0.001] on the three different types of eccentricity.

### 3.2. Result III: EEG Topographical and Time-Frequency Analysis

**Figure 6:**
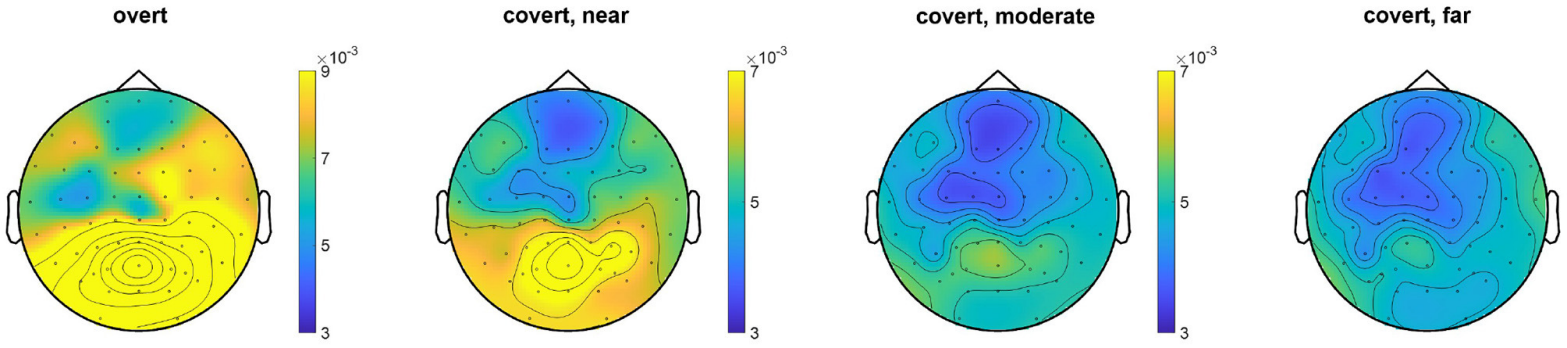
Topographic map comparison for the overt attention condition and different levels of covert attention across all trials for each subject. The color represents the value of the power spectrum. The SSVEP amplitudes were standardized to z scores before computing grand averages to address intersubject variability.

The EEG topography result for the average of all subjects under the overt condition and different levels of covert attention can be compared in Figure 6. When subjects directed their attention covertly to the visual target rather than to the fixation cross, the SSVEP amplitudes at the stimulus frequency decreased over the area near the occipital and parietal lobes [*F*_(1,48)_ = 22.43, *p* < 0.0001]. Additionally, covert attention with the flickering target further from the center cross had a narrower power distribution than that with targets closer to the cross. A significant main effect of the three groups [*F*_(2,72)_ = 3.62, *p* = 0.032] over the three different types of covert attention was found.

**Figure 7:**
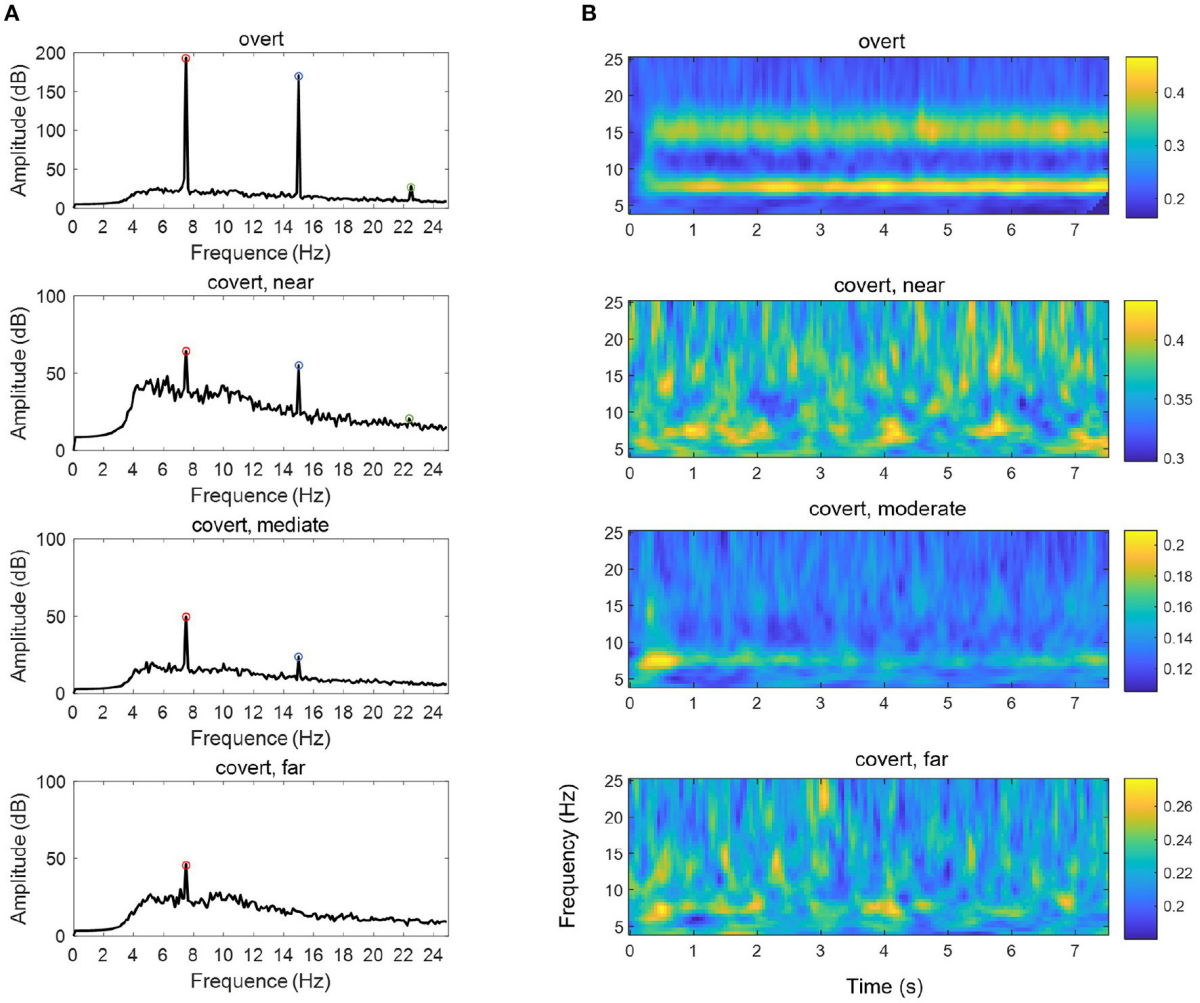
The mean PSD **(A)** and ITPC **(B)** of an SSVEP under the overt condition and different types of covert attention. The data were averaged over all 23 selected channels (see section 2.2) and across all subjects. The red circles, blue circles and green circles in **(A)** indicate the fundamental frequency of 7.5 Hz, the second-harmonic frequency of 15.0 Hz and the third-harmonic frequency of 22.5 Hz, respectively.

We also compared the difference in EEG power across time-frequency windows between these two experiments, as shown in Figure 7A. The data were standardized per channel of each trial, and outliers were removed before the frequency and time-frequency analysis. In the spectral domain, it can be seen that the amplitude of the fundamental frequency, as well as the second-harmonic frequency and the third-harmonic frequency in overt attention, was greater than each of that in different covert attention [*F*_(1,48)_ = 56.59, *p* < 0.0001]. For the details of covert attention, the amplitude at the three frequencies of interest showed a decreasing trend from the near condition to the far condition [*F*_(2,72)_ = 2.71, *p* = 0.073 at 7.5 Hz, *F*_(2,72)_ = 13.38, *p* < 0.001 at 15.0 Hz, *F*_(2,72)_ = 20.65, *p* < 0.001 at 22.5 Hz].

In addition, compared with that in the overt condition, the ITPC in the covert condition (Figure 7B) was reduced [*F*_(1,48)_ = 20.77 ~ 74.01, *p* < 0.0001 at 7.5 Hz, *F*_(1,48)_ = 41.88 ~ 143.81, *p* < 0.0001 at 15.0 Hz]. Additionally, a significant main effect of the three groups over the three different types of covert attention across the whole was found [*F*_(2,72)_ = 20.77 ~ 45.80, *p* < 0.0001 at 7.5 Hz, *F*_(2,72)_ = 30.28 ~ 88.50, *p* < 0.0001 at 15.0 Hz].

### 3.3. Result III: Neural Signatures

**Figure 8:**
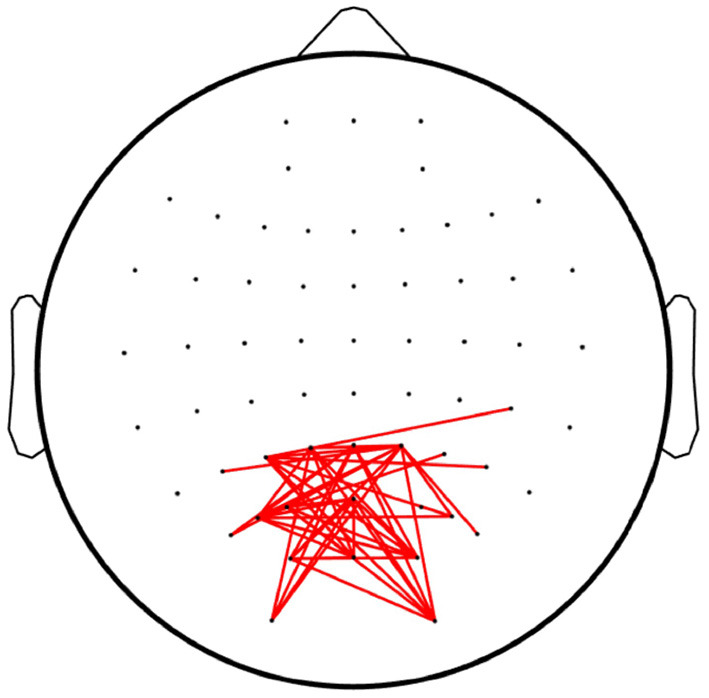
Linkages with significant differences among the overt attention and covert attention conditions. The red lines denote the edges with significant differences [*p* < 0.05, false discovery rate (FDR)-corrected for multiple comparisons].

Figure 8 depicts the linkages with significant differences (*p* < 0.05) among the overt and covert attention conditions for all subjects as revealed by paired-sample *t*-test. The results show that significantly different connections mainly occurred between the nodes located in the occipital lobes. However, no significant differences were found in the linkages between the different groups of covert attention, i.e., between near and moderate, between near and far, and between moderate and far. This conclusion is consistent with an fMRI study (Beauchamp et al., 2001) that revealed that more activity was observed in the precentral sulcus during overt attention than during covert attention. Another finding of this study is similar to that of the aforementioned fMRI study (Liu et al., 2003): when the subjects tended to pay attention to peripheral stimuli, the active network did not extend into the superior frontal or inferior frontal areas of the brain.

### 3.4. Result III: Classification Performance

**Figure 9:**
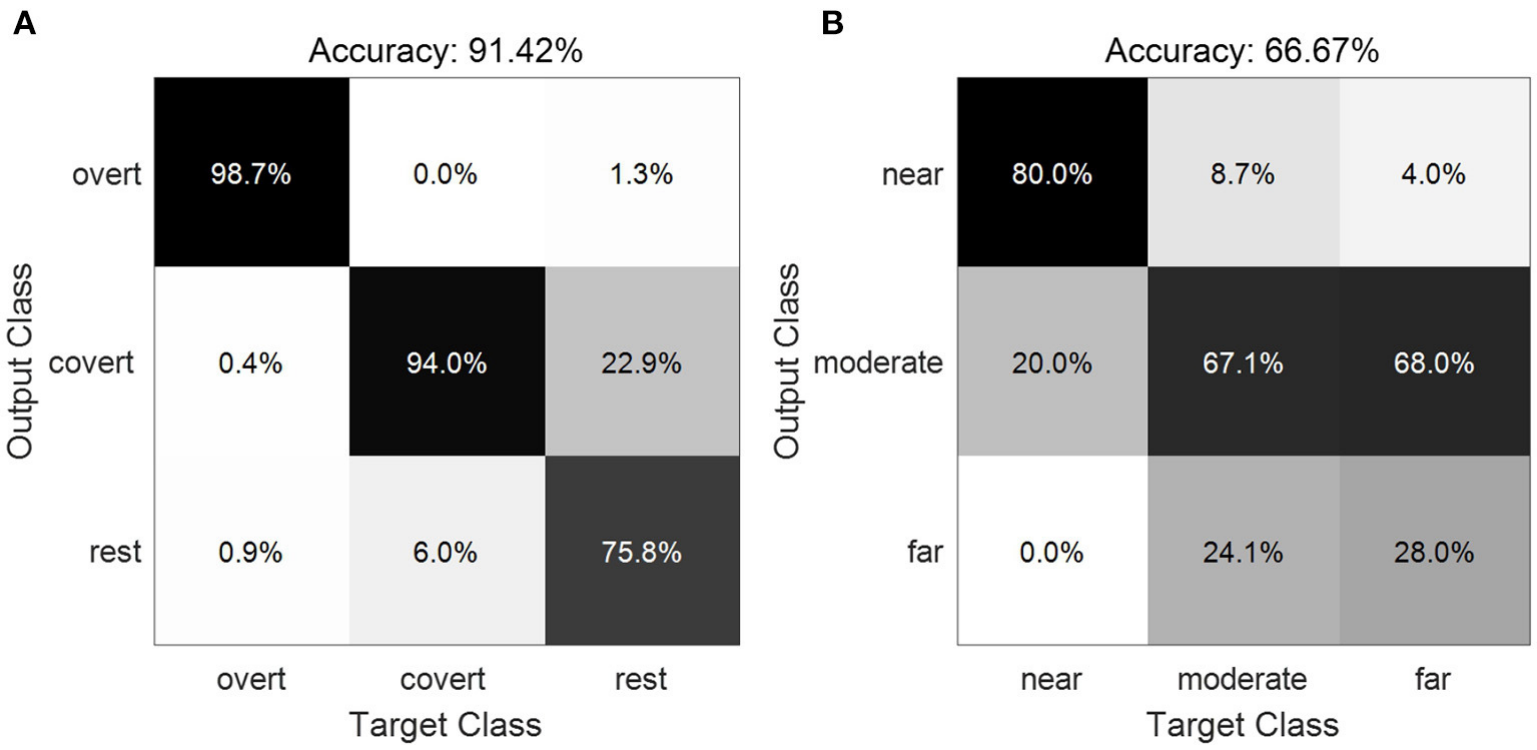
Confusion matrix showing the classification results of the three classes between the overt, covert and no attention **(A)** and of the three types of covert attention **(B)**.

Figure 9 displays the confusion matrices averaged across subjects. The diagonal cells show the number of cases that were correctly classified, and the off-diagonal cells show the misclassified cases. In total, the average accuracy of 91.42±5.30% for the three classes between the overt, covert and no attention (Figure 9A) was obtained across all subjects, as entries on the main diagonal indicate that the most prominent features were identified. Large values were also observed on the superdiagonal and subdiagonal lines between covert attention and unattended attention, suggesting that these two conditions are relatively easily misclassified as each other. As for the decode performance of different types of covert attention, we acquired a average accuracy of 66.67±14.21% across all subjects (Figure 9B). Values on the main diagonal demonstrated that the most prominent features for the types of near and moderate condition were recognized except the type of far condition as most trails were misclassified into the moderate condition.

## 4. Discussion

In this paper, we explored the influence of target eccentricity on the EEG-based covert attention and assessed the differences between different levels of covert attention and overt attention. The visual search type (e.g., the flickering circle in this study) and set size (e.g., the diameter of the circle) were fixed for all subjects to examine the influence of a single factor (i.e., the eccentricity effect) on the visual attention process. The extended CCA-based relevance analysis (FBCCA) was used to calculate parameters for evaluating subjects brain reaction in the above attention tasks. Furthermore, the multiple EEG measurements provided more details on the variance in their attention when faced with visual targets of different eccentricities. Our findings have quantified the eccentricity effect, as visual attention tends to be better when the target is presented more central to the fovea and worse when the target is further in the periphery of the retina (Carrasco et al., 1995; Carrasco and Barbot, 2014).

The cortical magnification factor (CMF) and receptive field (RF) size are well-recognized as two fundamental properties of the visual cortex during selective attention (Van Essen et al., 1984; Carrasco, 2011; Harvey and Dumoulin, 2011). The CMF indicates that more neurons process the central visual field than the peripheral parts (Harvey and Dumoulin, 2011), and the RF describes the visual field region where stimulation elicits a response. Figures 3–5 reveal that regardless of the type of distance used to characterize the eccentricity (Euclidean distance, horizontal distance and vertical distance), the correlation parameter of attention showed a decreasing tendency as eccentricity increased. This might be explained by the fact that the number and density of RFs tend to decrease and the spacing among them tends to increase with eccentricity (De Valois and De Valois, 1980; Carrasco, 2011; Zhaoping and Li, 2014). Additionally, the neurons associated with the fovea have smaller RF sizes than the peripheral neurons, making it easier to elicit powerful oscillations via central visual stimulation than via covert attended stimulation.

In terms of scalp topography, a greater proportion of the cortex was devoted to processing input from the central part of the visual field than the periphery due to the CMF (i.e., the energy distribution was focused more over the occipital region during overt attention than during covert attention). This finding is consistent with the conclusion of other studies highlighting that as eccentricity increases, information is pooled over a larger cortical area, diminishing sensitivity to fine patterns (Carrasco and Barbot, 2014; Staugaard et al., 2016). In addition to the difference in activation patterns among the overt and covert conditions shown in Figures 6, 7, we could also observe the presence of denser network linkages (Figure 8) in the visual sensory cortices (i.e., occipital lobes). In other words, only weaker information propagation and interactions between the areas of occipital lobes could be observed if the processing of covert attention was treated as circuits than brain region considered in isolation.

**Figure 10:**
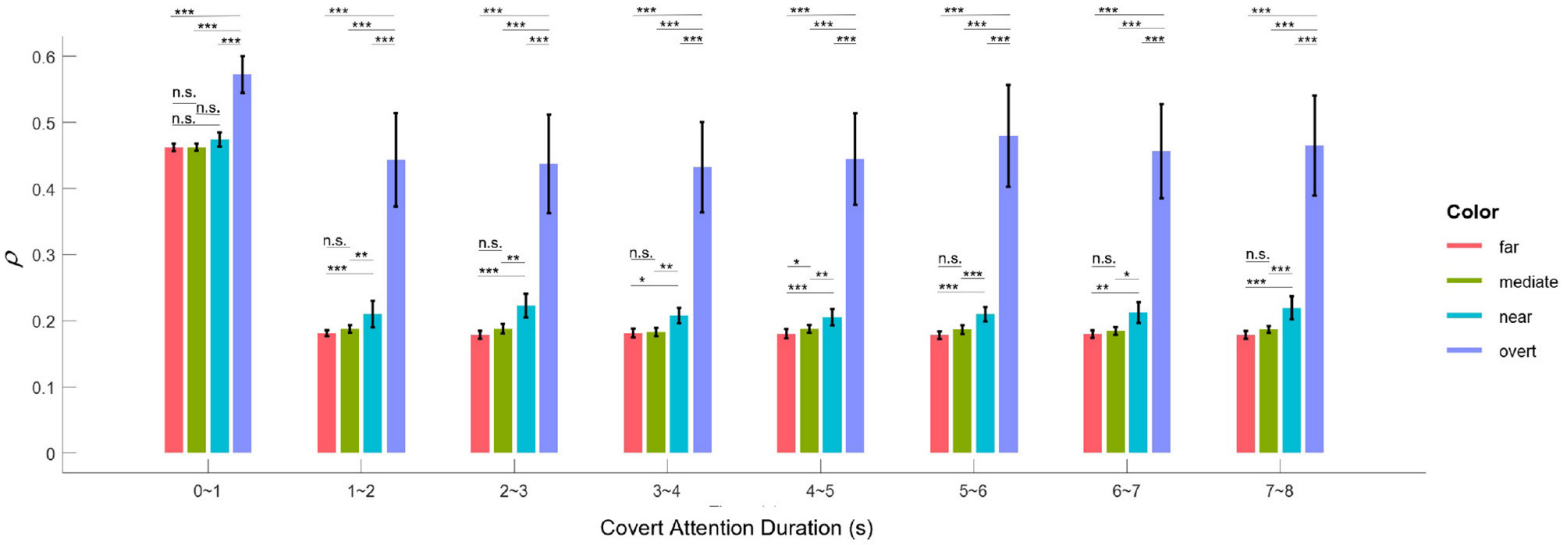
Mean correlation parameter of attention (***ρ***) for the overt and the four covert conditions across all subjects for different lengths of time. The black asterisk * denotes that the attention response of the condition with the higher ***ρ***-value is significantly more pronounced than that with the lower ***ρ***-value at the 0.05 level, and ** and *** indicate likewise for the 0.01 and 0.001 levels, respectively. Error bars show standard errors of the means.

The application of SSVEPs to overt and covert attention demonstrated that attentional shifts can be periodically manipulated by the frequency of the underlying oscillations. This idea is in line with two previous studies (Buschman and Miller, 2009; Busch and VanRullen, 2010), although they used different frequencies or frequency bands (7.5 Hz in our case, 18–34 Hz in Buschman and Miller, 2009 and 7–10 Hz in Busch and VanRullen, 2010). Furthermore, the results shown in Figure 7B also reveal that the facilitative effect of covert attention endures as long as this attention is directed to a specific location. Therefore, the evidence we presented supports another conclusion regarding selective attention: it can also be a sustained via voluntary operation, known as “endogenous” attention, as previously demonstrated by many pieces of behavioral evidence (Carrasco, 2011; Carrasco and Barbot, 2014). Moreover, the EEG response to visual attention varied across stimulation durations. Figure 10 shows the variation in the intensities of overt attention as well as different levels of covert attention as the subjects' fixation time increased. The brain response in the overt and covert attention conditions stabilized after the first second, with the former showing much greater value than the latter. In terms of the different covert attention conditions, significant differences were found between the near and moderate and between the near and far conditions but not between the moderate and far conditions (*p* > 0.05). It is worth noting that the critical eccentricities (3° and 10°) were determined to maximize the discrimination in different conditions through a grid search method. Nevertheless, this statistical results still suggest that the brain response in the covert attention tasks did not vary homogeneously with increasing eccentricity, as visual stimuli located too far from the center of vision contributed less to the enhancement in spatial resolution.

In this study, the influence of another specific aspect of covert attention (i.e., stimulus orientation: left vs. right, horizontal vs. vertical) was investigated at isoeccentric locations. Comparing Figure 3 vs. Figures 4, 5 reveals better spatial resolution along the Euclidean distance than along the horizontal distance [*F*_(1,48)_ = 9.52, *p* < 0.01] or vertical distance [*F*_(1,48)_ = 25.93, *p* < 0.001], respectively. Furthermore, Figures 4, 5 demonstrate that most subjects showed more noticeable response when shifting their attention to horizontal targets than to vertical targets [*F*_(1,48)_ = 7.62, *p* < 0.01]. This finding supports the hypothesis that spatial resolution is better along the horizontal meridian than along the vertical meridian (Fuller et al., 2008; Montaser-Kouhsari and Carrasco, 2009; Carrasco, 2011). However, we did not observe the same lateralization pattern mentioned in other studies (Bahramisharif et al., 2011; Roijendijk et al., 2013), as no significant difference was found in the SSVEP response between covert targets appearing on the left or right side of the screen [*F*_(1,48)_ = 0.002 ~ 2.38, *p* > 0.05].

The classification performance in Figure 9A shows that the use of covert attention can provide a new paradigm for BCI systems even with a single stimulus. The addition of adding no attention trials into categorization analysis demonstrated that these BCI systems can run at an asynchronous mode, in which users choose to send a command at their discretion or to remain in the idle state. Furthermore, the acquired accuracy as shown in Figure 9B indicates these BCI systems have the potential of identifying the retinal eccentric level of covert attention with the limitation of deploying the eccentricity within an appropriate range. The poor performance of distinguishing the far condition indicates that the spatial resolution might decreases as visual acuity might be insufficient for the target's identification while the peripheral stimulation is too far from the central cross (Carrasco, 2011; Carrasco and Barbot, 2014). Nevertheless, the ability to decode the attention state could support optimization of the locus information of the stimulus in alerting applications. People, especially those who are completely paralyzed and have difficulty fixing their gaze, could choose an item simply by paying attention to it, as many existing BCI systems have important dependencies on gaze (Brunner et al., 2010; Kaufmann et al., 2011).

## 5. Conclusions

In this study, we showed that the processing of covert attention with SSVEP stimulation is quantifiably and negatively affected by increasing eccentricity. The comparison between overt and covert attention confirmed the hypothesis that covert attention enhances spatial resolution. The decoding performance also confirmed that the subjects' visual attention status (overt attention, covert attention, or no attention) can be successfully distinguished in SSVEP-based attention paradigms, which may contribute to the asynchronous control for BCI systems.

## Data Availability Statement

The raw data supporting the conclusions of this article will be made available by the authors, without undue reservation.

## Ethics Statement

The studies involving human participants were reviewed and approved by Ethics Committee of Sichuan Provincial Rehabilitation Hospital. The patients/participants provided their written informed consent to participate in this study.

## Author Contributions

TY, YL, and YZ conceived of the main idea of this study. YZ realized it, completed the experiments with LH, and wrote the paper with help from TY and YL. YZ and TY also built the covert attention paradigm and performed the data processing. All authors contributed to the article and approved the submitted version.

## Funding

This work was supported by the Key R&D Program of Guangdong Province, China under Grant 2018B030339001, the National Natural Science Foundation of China under Grant 61633010, and the Key Realm R&D Program of Guangzhou, China under Grant 202007030007.

## Conflict of Interest

The authors declare that the research was conducted in the absence of any commercial or financial relationships that could be construed as a potential conflict of interest.

## Publisher's Note

All claims expressed in this article are solely those of the authors and do not necessarily represent those of their affiliated organizations, or those of the publisher, the editors and the reviewers. Any product that may be evaluated in this article, or claim that may be made by its manufacturer, is not guaranteed or endorsed by the publisher.
